# Mitogenesis of Vascular Smooth Muscle Cell Stimulated by Platelet-Derived Growth Factor-bb Is Inhibited by Blocking of Intracellular Signaling by Epigallocatechin-3-*O*-Gallate

**DOI:** 10.1155/2013/827905

**Published:** 2013-11-06

**Authors:** Mi Hee Lee, Byeong-Ju Kwon, Min-Ah Koo, Kyung Eun You, Jong-Chul Park

**Affiliations:** ^1^Cellbiocontrol Laboratory, Department of Medical Engineering, Yonsei University College of Medicine, 134 Shinchon-dong, Seodaemun-gu, Seoul 120-752, Republic of Korea; ^2^Brain Korea 21 PLUS Project for Medical Science, Yonsei University, 134 Shinchon-dong, Seodaemun-gu, Seoul 120-752, Republic of Korea

## Abstract

Epigallocatechin gallate (EGCG) is known to exhibit antioxidant, antiproliferative, and antithrombogenic effects and reduce the risk of cardiovascular diseases. Key events in the development of cardiovascular disease are hypertrophy and hyperplasia according to vascular smooth muscle cell proliferation. In this study, we investigated whether EGCG can interfere with PDGF-bb stimulated proliferation, cell cycle distribution, and the gelatinolytic activity of MMP and signal transduction pathways on RAOSMC when it was treated in two different ways—cotreatment with PDGF-bb and pretreatment of EGCG before addition of PDGF-bb. Both cotreated and pretreated EGCG significantly inhibited PDGF-bb induced proliferation, cell cycle progression of the G0/G1 phase, and the gelatinolytic activity of MMP-2/9 on RAOSMC. Also, EGCG blocked PDGF receptor-*β* (PDGFR-*β*) phosphorylation on PDGF-bb stimulated RAOSMC under pretreatment with cells as well as cotreatment with PDGF-bb. The downstream signal transduction pathways of PDGFR-*β*, including p42/44 MAPK, p38 MAPK, and Akt phosphorylation, were also inhibited by EGCG in a pattern similar to PDGFR-*β* phosphorylation. These findings suggest that EGCG can inhibit PDGF-bb stimulated mitogenesis by indirectly and directly interrupting PDGF-bb signals and blocking the signaling pathway via PDGFR-*β* phosphorylation. Furthermore, EGCG may be used for treatment and prevention of cardiovascular disease through blocking of PDGF-bb signaling.

## 1. Introduction

Several vascular diseases involve vascular smooth muscle cell (VSMC) proliferation as their primary mechanism. Dedifferentiated VSMCs induce cell proliferation and migration, as well as extracellular matrix (ECM) protein deposition [1–4]. Intimal hyperplasia is an excessive tissue ingrowth and chronic structural lesion that can be observed at the site of atherosclerotic lesion formation, arterial angioplasty, vascular graft anastomoses, and so forth. This phenomenon is caused by the phenotype change of VSMCs from a differentiated state to a dedifferentiated one. Several studies have focused on VSMC phenotype switching, decreasing expression of smooth muscle specific contractile markers such as *α*-smooth muscle actin, smooth muscle myosin heavy chain, and calponin, [5, 6], migration and proliferation from media to the intima, and extracellular matrix remodeling [7, 8]. Vascular proliferation is the most important factor in intimal hyperplasia and is linked to other cellular processes such as migration, inflammation, and extracellular matrix production.

Platelet-derived growth factor-bb (PDGF-bb) is one of the most potent mitogens and chemoattractants for VSMC and plays a central role via simultaneous interactions between itself [9]. In previous studies, it was confirmed that PDGF-bb induced phenotype switching [10, 11], MMP-2 upregulation [12, 13], and migration [14] on VSMCs. PDGF-bb is also known to bind to the PDGR receptor (PDGFR)-*β* and subsequently activates several intracellular signaling cascades, including the extracellular signal-regulated kinase (ERK), p38 mitogen-activated protein kinase (p38 MAPK) pathways, and phosphatidylinositol 3-kinase-Akt (PI3 K-Akt), and stimulates VSMC dedifferentiation [15]. 

Epigallocatechin gallate (EGCG) is the most prevalent polyphenol contained in green tea. This has been reported to have antioxidant, antiproliferative, and antithrombogenic effect. Recent experiments have suggested that green tea catechins can reduce atherosclerotic lesions in various animal models and prevent cardiovascular diseases [16–18]. In addition, EGCG inhibits VSMC invasion by preventing matrix metalloproteinase (MMP) expression and provides a protective effect against atherosclerosis and cancer via matrix degradation [19].

In this study, we investigated the effects of EGCG on proliferation, cell cycle, and the intracellular signal transduction pathway of PDGF-bb in rat aortic vascular smooth muscle cell (RAOSMC) and demonstrated the preventive mechanism of PDGF-bb stimulated RAOSMC dedifferentiation.

## 2. Materials and Methods

### 2.1. Cell Culture

Rat aortic smooth muscle cells (RAOSMC) were purchased from Biobud (Seoul, Republic of Korea), and cells at passage 5 to 9 were used. The cells were routinely maintained in Dulbecco's Modified Eagle Medium (Gibco, Carlsbad, CA, USA) and supplemented with 10% fetal bovine serum (Sigma, St. Louis, MO, USA) and a 1% antibiotic-antimycotic solution containing 10,000 units penicillin, 10 mg streptomycin, and 25 *μ*g amphotericin B per mL (Sigma) at 37°C in a humidified atmosphere of 5% CO_2_. 

### 2.2. Cell Stimulation by PDGF-bb

EGCG (Teavigo), the major polyphenolic constituent of green tea, was purchased from DSM Nutritional Products Ltd. It was dissolved in 50% DMSO (Sigma) for a stock solution of 100 mM and then diluted to the desired concentrations with media prior to cell treatment. For the experiments, RAOSMCs were routinely incubated. Cells were synchronized in serum-free medium for 24 h before experiments. Prior to the experiments, the cells were incubated with two different methods. With the first method, the synchronized RAOSMCs were preincubated with EGCG in serum-free media for 24 h. Then, EGCG-treated cells were washed twice with PBS and stimulated with serum-free media containing 10 ng/mL of human recombinant PDGF-bb (Sigma) for a desired length of time. For the second method, cells were synchronized in serum-free DMEM medium for an additional 24 h and stimulated with 10 ng/mL PDGF-bb and soluble EGCG.

### 2.3. Cell Proliferation and DNA Synthesis

Cell proliferation was determined by MTT assay (reduction of 3-(4,5-dimethylthiazol-2-yl)-2,5-diphenyltetrazolium bromide to a purple formazan product, Sigma) and a 5-bromo-2′-deoxyuridine (BrdU) incorporation assay (Roche Applied Science, Seoul, Republic of Korea). 

For the MTT assay, the cells incubated with 0.5 mg/mL of MTT in the last 4 h of the culture period were tested at 37°C in the dark. The media were decanted, and the produced formazan salts weredissolved with dimethylsulphoxide, and absorbance was determined at 570 nm by an automatic microplate reader (Spectra Max 340, Molecular Devices Co., Sunnyvale, CA, USA). 

For BrdU incorporation assay, BrdU-labeling solution was added to the cells, and it was reincubated for 2 h at 37°C. Labeling medium was then removed, and the cells were incubated with fixated solution for 30 min at room temperature. After fixation of the cells, anti-BrdU-POD working solution was added, and the cells were incubated for 90 min at room temperature. Then, the substrate solution was added, and absorbance was measured at 370 nm with 492 nm reference wavelength by an automatic microplate reader (Spectra Max 340, Molecular Device Co.) 

### 2.4. Cell Cycle Analysis

To analyze the cell cycle, RAOSMCs were collected and washed with cold phosphate-buffered saline (PBS, pH 7.2). The cells were resuspended in 95% cold methanol for 1 h at 4°C and then centrifuged at 120 ×g for 5 min. The resultant pellet was washed twice with cold PBS and incubated with RNase A (20 U/mL final concentration, Sigma) at 37°C for 30 min. Intracellular DNA was labeled with 100 *μ*g/mL propidium iodide (PI, Sigma) for 1 h and then analyzed with a fluorescence-activated cell sorter (FACSCalibur, Becton Dickinson, San Jose, CA, USA). The cell cycle profile was gained by analyzing at least 20,000 cells with the ModFit LT program written by Mac-App (Becton Dickinson).

### 2.5. Gelatin Zymography

Gelatinase activity was detected in the conditioned medium of cultured RAOSMC. The conditioned media mixed with Laemmli buffer under nonreducing conditions were loaded onto 10% SDS-polyacrylamide gel containing 0.1% gelatin. After electrophoresis, the gels were washed for 20 min at room temperature in 2.5% Triton X-100 and incubated for 18 h at 37°C with reaction buffer (50 mM Tris base (pH7.6), 0.2 M NaCl, 5 mM CaCl_2_, 0.02% Brij 35). The gels were stained with Coomassie Brilliant Blue R-2500 (0.1%) and destained. Densitometric analysis was performed with imageJ software (National Institutes of Health, Bethesda, MD, USA).

### 2.6. Western Blot Analysis

After being stimulated with PDGF-bb, the cells were washed twice with cold PBS (10 mM, pH 7.4). Ice-cold RIPA lysis buffer (Santa Cruz Biotechnology Inc., Santa Cruz, CA, USA) was added to the cells and incubated for 5 min. The cells were scraped, and the lysate was cleared by centrifugation at 14,000 ×g for 20 min at 4°C. The resultant supernatant (total cell lysate) was collected. Protein concentration was determined by using a DC Bio-Rad assay kit (Bio-Rad Laboratories Inc., Hercules, CA, USA). For immunoblot analysis, the protein was run on SDS-PAGE and then electrotransferred onto a PVDF membrane. The membrane was blocked with the buffer (5% nonfat dry milk and 1% Tween-20 in 20 mM TBS, pH 7.6) for 1 h at room temperature and then probed overnight with phospho-PDGFR-*β* (p-PDGFR-*β*), PDGFR-*β*, phospho-MEK1/2 (p-MEK1/2), MEK1/2, phospho-p42/44 MAPK (p-p42/44 MAPK), p42/44 MAPK, phospho-Akt (p-Akt), Akt, phospho-p38 MAPK (p-p38 MAPK), and p38 MAPK used at a 1 : 1,000 dilution from Cell Signaling Technology (Danvers, MA, USA). Detection of horseradish peroxidase-conjugated secondary Ab (e.g., anti-rabbit IgG (1 : 5,000) and anti-mouse IgG (1 : 2,000) from Santa Cruz Biotechnology Inc.) was accomplished using enhanced chemiluminescence using the ECL Plus detection kit (Amersham Biosciences, Buckinghamshire, England). Densitometric analysis was performed with imageJ (National Institutes of Health, Bethesda, MD, USA).

### 2.7. Statistical Analysis

All variables were tested in three independent cultures for each experiment. The results are reported as a mean ± SD and compared to non-treated controls. Statistical analysis was performed using a one-way (ANOVA), followed by a Tukey HSD test for multiple comparisons using SPSS software. A *P* value of <0.05 was considered statistically significant.

## 3. Results

### 3.1. Inhibitory Effect of Proliferation by PDGF-bb on EGCG Pretreated RAOSMC

**Figure 1:**
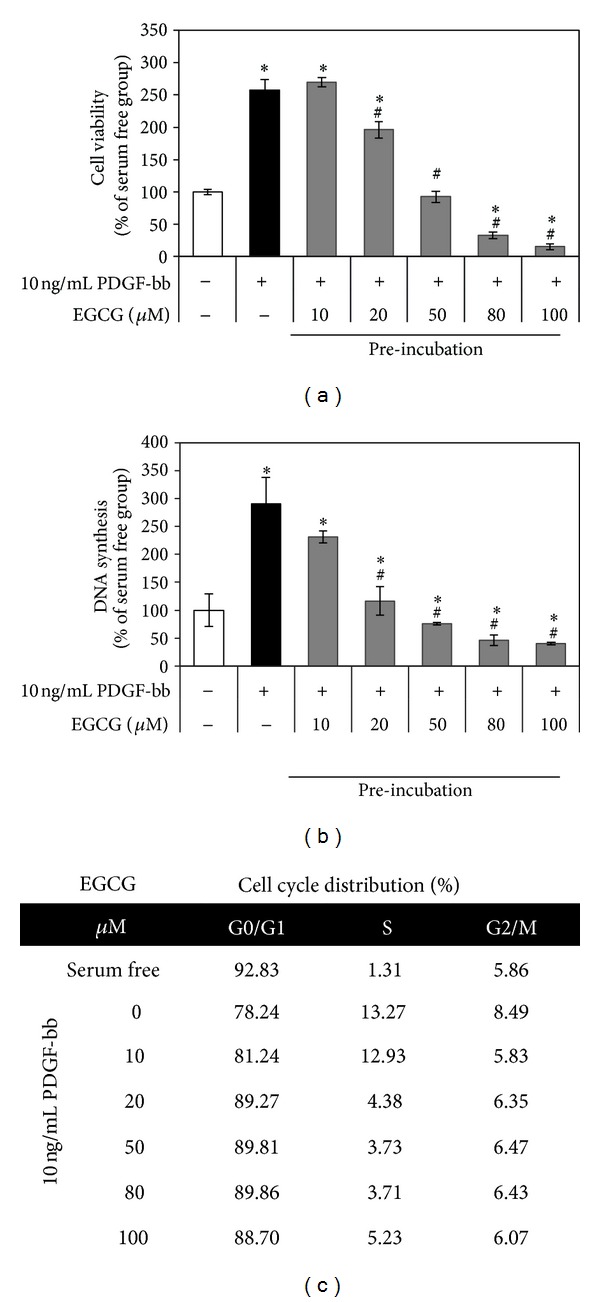
The antiproliferative activity and cell cycle arrest activity by PDGF-bb on EGCG preincubated RAOSMC. After 24 h of starvation with DMEM containing increasing concentrations (10–80 *μ*M) of EGCG, cells at 80% confluence were washed and treated with 10 ng/mL PDGF-bb. (a) The effects of growth inhibition on PDGF-bb stimulation in EGCG preincubated RAOSMC. Cell viability was detected using the MTT assay. *P < 0.05 compared with nonstimulation control; ^#^ *P* < 0.05 compared with the 10 ng/mL PDGF-bb stimulated control. (b) The effect of EGCG preincubation on PDGF-bb-induced DNA synthesis in RAOSMC. DNA synthesis was detected using the BrdU incorporation assay. *P < 0.05 compared with nonstimulation control; ^#^ *P* < 0.05 compared with 10 ng/mL PDGF-bb stimulated control. (c) EGCG preincubation with PDGF-bb stimulated cell cycle distribution in RAOSMC. Cell cycle distribution was determined by propidium iodide (PI) labeling followed by flow cytometry. The percentages of cells in the G0/G1, S, and G2/M phases were calculated using Modifit computer software and represented within the histograms.

To investigate proliferation by PDGF-bb stimulation on RAOSMC pretreated with EGCG, increasing EGCG concentration was treated with serum-free DMEM for 24 h at 70~80% confluence RAOSMC. Cells were then washed twice with PBS and incubated with 10 ng/mL PDGF-bb for 24 h. 10 ng/mL PDGF-bb induced a significant (*P* < 0.05) RAOSMC proliferation as compared to the nonstimulated group as assessed by increased DNA synthesis and increased formazan absorbance. When cells were preincubated with increasing concentrations of EGCG, cell proliferation by 10 ng/mL PDGF-bb was significantly (*P* < 0.05) decreased in a dose-dependent manner of EGCG. Therefore, cell viability (Figure 1(a)) and DNA synthesis (Figure 1(b)) were not significantly affected in concentrations up to 50 *μ*M. To investigate the effects of EGCG pretreatment on cell cycle distribution, DNA cell cycle analysis was performed on RAOSMC stimulated with PDGF-bb. As shown in Figure 1(c), EGCG pretreatment resulted in an appreciable increase in cells in the G0/G1phase, with a decrease in S-phase cells in up to 20 *μ*M EGCG pretreatment. These results indicate that EGCG pretreatment can suppress cell cycle progression and cell growth on RAOSMC with distributed PDGF-bb stimulation.

### 3.2. Inhibitory Effect of Proliferation by Cotreatment of PDGF-bb and EGCG on RAOSMC

**Figure 2:**
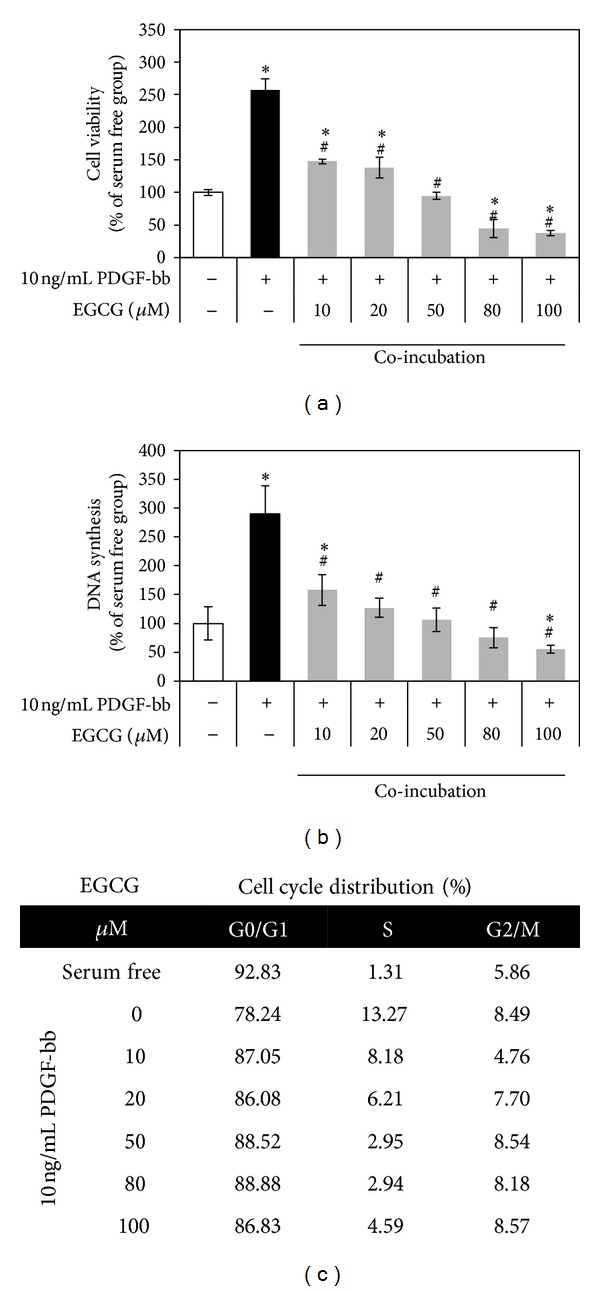
Antiproliferative activity and cell cycle arrest activity of PDGF-bb with EGCG on RAOSMC. After 24 h of starvation with serum-free DMEM, the cells were treated with 10 ng/mL PDGF-bb and increasing concentrations (10–80 *μ*M) of EGCG for 24 h. (a) The effects of EGCG growth inhibition on PDGF-bb stimulation in RAOSMC. Cell viability was detected using an MTT assay. *P < 0.05 compared with nonstimulation control; ^#^ *P* < 0.05 compared with the 10 ng/mL PDGF-bb stimulated control. (b) The effects of EGCG on PDGF-bb-induced DNA synthesis in RAOSMC. DNA synthesis was detected using the BrdU incorporation assay. *P < 0.05 compared with nonstimulation control; ^#^ *P* < 0.05 compared with 10 ng/mL PDGF-bb stimulated control. (c) The effect of EGCG on PDGF-bb stimulated cell cycle distribution in RAOSMC. Cell cycle distribution was determined by propidium iodide (PI) labeling followed by flow cytometry. The percentages of cells in the G0/G1, S, and G2/M phases were calculated using Modifit computer software and represented within the histograms.

To investigate proliferation by PDGF-bb stimulation with EGCG on RAOSMC, synchronized cells were incubated for 24 h with increasing concentrations of EGCG and 10 ng/mL PDGF-bb. Cotreatment with EGCG and PDGF-bb significantly inhibited the proliferation of RAOSMC by PDGF-bb stimulation (Figure 2(a)). Similarly, Figure 2(b) shows that DNA synthesis is also inhibited. According to BrdU incorporation into RAOSMC, cotreatment of EGCG (10 *μ*M) and PDGF-bb represented more inhibitory effects than pretreatment of EGCG. Proliferation was completely inhibited at a concentration of 50 *μ*M EGCG. EGCG induced a significant accumulation of the cells in the G0/G1 phase of the cell cycle at up to 10 *μ*M. Inhibition of cell growth in RAOSMC may be caused by G0/G1 arrest as EGCG interrupts PDGF-bb stimulated cell cycle progression.

### 3.3. Preventive Effect of Active MMP-2/9 Production by EGCG on PDGF-bb Stimulated RAOSMC

**Figure 3:**
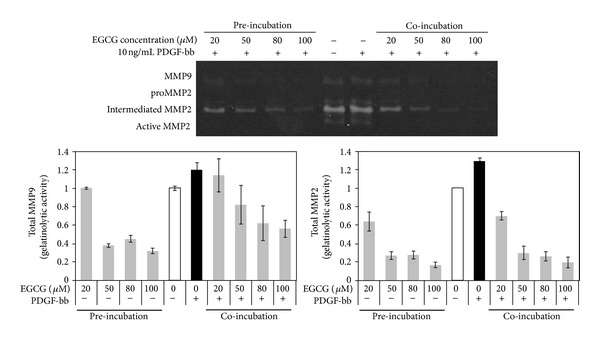
Inhibitory effect of EGCG on PDGF-bbinduced MMP gelatinolytic activity in RAOSMC. Gelatin catalytic activity was analyzed by gelatin zymography using conditioned medium. The band intensity was normalized by densitometry. PDGF-bb induced the gelatinolytic activity of MMP-9 and MMP-2. However, both preincubated and coincubated EGCG inhibited secretion of PDGF-bbinduced MMP-9 and MMP-2 activity.

MMP-2 and MMP-9 were detected in the conditioned media from cultured RAOSMC for 24 h with EGCG and PDGF-bb by gelatin zymography assay. After stimulation with PDGF-bb, RAOSMC showed more pro-MMP conversion into the intermediated and active form of MMP-2, and increased the MMP-9 release. As shown in Figure 3, EGCG pretreated RAOSMC significantly reduced the PDGF-bb-induced gelatinolytic activities of active MMP-2 and MMP-9. Therefore, the stimulatory effect of PDGF-bb also caused a reduction in MMP-2/9 gelatinolytic activity in a concentration-dependent manner by treatment of RAOSMC with EGCG. The inhibitory effect of MMPs gelatinolytic activity was dose-dependently expressed on EGCG. The active form of MMP-2 was not detected at up to 20 *μ*M of EGCG.

### 3.4. Inhibitory Effect of PDGF-bb Stimulated Signal Transduction Pathway in EGCG Preincubated RAOSMC

**Figure 4:**
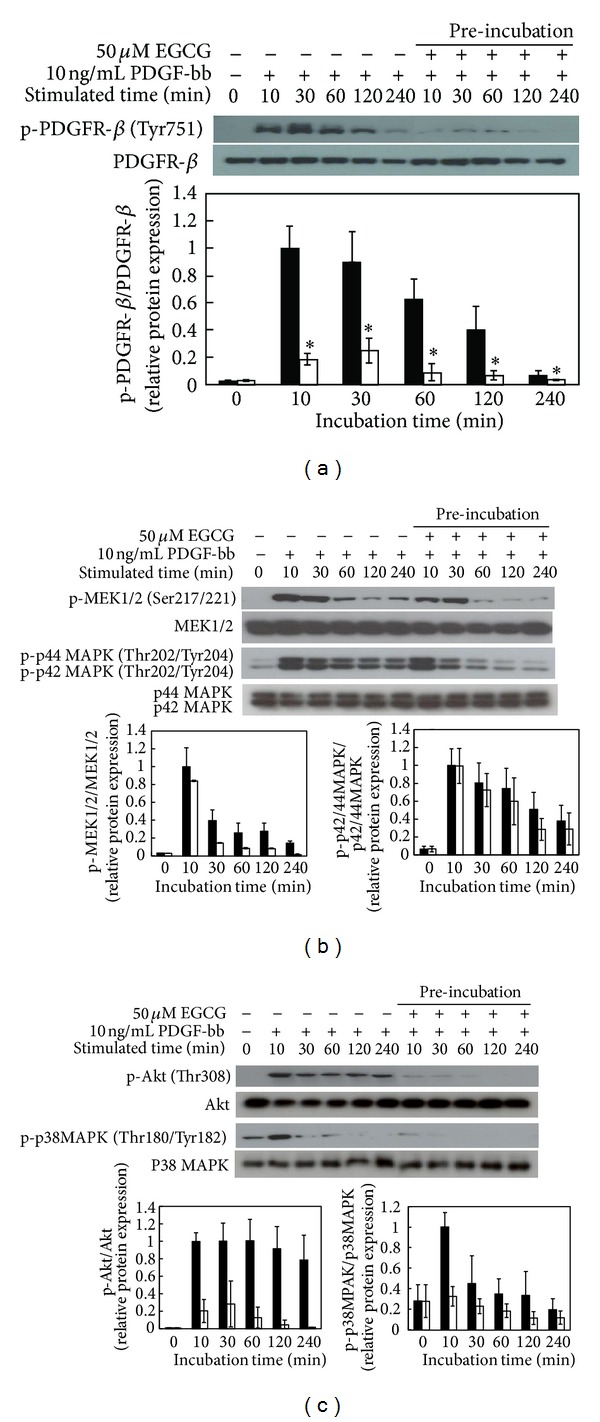
Modulation of PDGF-bb stimulatory signal pathways on EGCG preincubated RAOSMC. RAOSMC preincubated with EGCG was stimulated with 10 ng/mL PDGF-bb for the desired time (10 m, 30 m, 1 h, 2 h, and 4 h, resp.), lysed, and lysates were immunoblotted with antibodies. After densitometric quantification, data were each expressed as the mean ± SD from three independent experiments. The black bars indicate expression by PDGF-bb stimulation. The white bars indicate expression by PDGF-bb stimulation on EGCGpretreated RAOSMC. (a) The expression of phospho-PDGFR-*β*  (Tyr751) in a time-dependent manner. The band intensity was normalized to total PDGFR-*β* expression. (b) The expression of phospho-MEK1/2 (Ser217/221) and phospho-p42/44 MAPK (Thr202/Tyr204) in a time-dependent manner. The band intensity was normalized to total MEK1/2 and p42/44 MAPK expression. (c) The expression of phospho-Akt (Thr308) and phospho-p38 MAPK (thr180/Tyr182) in time-dependent manner. The band intensity was normalized to total Akt and p38 MAPK expression.

To define the effects of EGCG pretreatment on signaling pathways of PDGF-stimulated mitogenesis, already synchronized RAOSMCs were incubated with EGCG and serum-free media for 24 h. For PDGF-bb stimulation, the cells were washed using PBS to remove EGCG, incubated for the desired time, and examined for levels of various proteins by Western blot analysis. Addition of 10 ng/mL PDGF-bb to serum-starved RAOSMCs led to complete PDGFR-*β* phosphorylation, which reached the peak within 10 min and then decreased to nearly baseline levels at 240 min. However, pretreated EGCG suppressed PDGFR-*β* phosphorylation by PDGF-bb and sustained only baseline level (Figure 4(a)). The phosphorylations of MEK1/2 and p42-44MAPK, downstream proteins of PDGF-induced signaling, were significantly increased between 10 and 30 min and declined over the following 240 min. However, pretreated EGCG inhibited MEK1/2 and p42-44MAPK phosphorylations in a time-dependent manner, similar to PDGFR-*β* phosphorylation (Figure 4(b)). In the other intracellular signal pathways, phosphorylations of Akt and p38 MAPK were activated by PDGF-bb stimulation. However, the Akt and p38 MAPK phosphorylations induced by PDGF-bb were inhibited in RAOSMCs by being pretreated with EGCG (Figure 4(c)). These results suggest that EGCG can indirectly inhibit the phosphorylation of PDGFR-*β* by PDGF-bb.

### 3.5. Inhibitory Effect of Signal Transduction Pathway on RAOSMC by PDGF-bb Stimulation with EGCG

**Figure 5:**
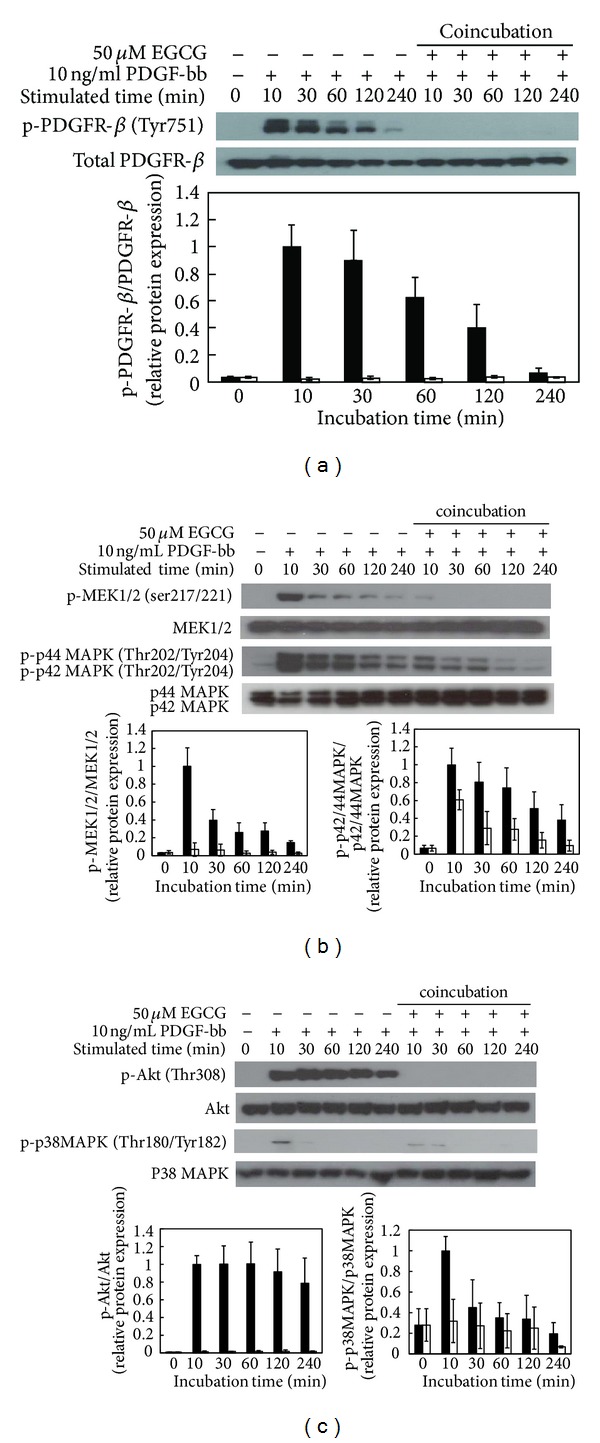
The effect of EGCG on modulation of PDGF-bb stimulatory signal pathways in RAOSMC. Serum-starved RAOSMC was stimulated with 10 ng/mL PDGF-bb and 50 *μ*M EGCG for the desired time (10 m, 30 m, 1 h, 2 h, and 4 h, resp.), lysed, and lysates were immunoblotted with antibodies. After densitometric quantification using the imageJ program, data were each expressed as the mean ± SD from three independent experiments. The black bar indicates expression by PDGF-bb stimulation. The white bar indicates expression by PDGF-bb stimulation with EGCG. (a) The expression of phospho-PDGFR-*β* (Tyr751) in a time-dependent manner. The band intensity was normalized to total PDGFR-*β* expression. (b) The expression of phospho-MEK1/2 (Ser217/221) and phospho-p42/44 MAPK (Thr202/Tyr204) in a time-dependent manner. The band intensity was normalized to total MEK1/2 and p42/44 MAPK expression. (c) The expression of phospho-Akt (Thr308) and phospho-p38 MAPK (Thr180/Tyr182) in a time-dependent manner. The band intensity was normalized to total AKt and p38 MAPK expression.

To characterize the signaling pathways by direct interaction between EGCG and PDGF-bb, serum-starved RAOSMCs were incubated with EGCG and PDGF-bb for the desired times. PDGFR-*β* phosphorylation was completely suppressed and inactivated on PDGF-bb induced RAOSMC by EGCG compared with the PDGF-stimulated samples that were processed on the same blot (Figure 5(a)). Therefore, MEK1/2 and p42/44 MAPK phosphorylations were suppressed and sustained at baseline levels by being cotreated EGCG with PDGF-bb (Figure 5(b)). The phosphorylations of Akt and p38 MAPK were also suppressed by inhibition of PDGF-bb signaling by EGCG. These results reveal that EGCG can directly interrupt PDGF-bb stimulation by inhibiting PDGFR-*β* phosphorylation.

## 4. Discussion

PDGF-bb is a major stimulator of VSMC dedifferentiation and is known to play a central role in the pathogenesis of various vascular disorders. Signal transduction pathways involve the activation of mitogen-activated protein kinases (MAPKs) on PDGF-induced responses. MAPK is a family of serine/threonine protein kinases with 3 subfamilies named c-jun-N-terminal kinase 1/2 (JNK1/2), ERK1/2, and p38 MAPK. PDGF stimulated rapid and significant activation of Akt, ERK1/2, and p38 MAPK in cultured VSMC. MAPKs are proposed to play a major role in the activation of various transcription factors [20, 21]. PDGF-bb binds with PDGFR-*β* and triggers receptor dimerization and autophosphorylation at tyrosine residues that activate the kinase and serve as recruitment sites for SH2 domain-containing proteins. Within minutes, many signaling modules are engaged, including Ras, Src, phosphoinositide 3′-kinase (PI3 K), SHP2, and phospholipase C*γ* (PLC*γ*) [9, 22, 23]. Downstream signals then activate PI3-K/PKB (Akt) and two MAPK pathways [24]. VSMC dedifferentiation is determined by activation of Akt pathway, p42/44 MAPK, and p38 MAPK pathways. Ultimately, this results in VSMC dedifferentiation via the recruitment, and activation of specific signaling pathway may mediate the migration and proliferation of VSMCs in response to injury such as the development of atherosclerosis and hypertension. Several studies have revealed that PDGFR targeted by synthetic tyrosine kinase inhibitors and antisense treatment reduce neointima formation in injured arteries [25, 26].

EGCG has been shown to have protective effects on the cardiovascular system, including antiatherosclerotic, antihypercholesterolemic, and antirestenosis effects [27–29]. Also, several studies have stated that EGCG inhibited proliferation, migration, and invasion of barrier by inhibition via intracellular signaling transduction pathway signals on VSMC stimulated with growth factor, such as angiotensin II [30, 31] and basic fibroblast growth factor (bFGF) [32]. A previous study showed that EGCG induced apoptosis of VSMCs in a p53- and NF-*κ*B-dependent manner [33, 34]. 

However, the dosage of polyphenols and flavonoids in cell culture studies may be much higher that than which occurs after oral administration in the body. The compounds may lose most of their functions after undergoing metabolism and circulation in vivo, but this may not be possible to evaluate in vitro. For that reason, concentration determined by in vitro experiment may be difficult to apply as a physiological dose to animals or humans [35–37]. Although not all cell culture findings are applicable for animal experiments, in vitro studies have provided important insights into the action mechanisms of flavonoids that would be physiologically achievable in human [37].

Polyphenolic catechins and flavonoids are generally safe and may possess beneficial properties for human health. Various clinical studies have revealed that they are effective at various organ sites [38]. However, unusually high dosage of natural products supplements may exhibit toxicity in vivo [36, 37]. Accordingly, numerous studies have been performed to improve the stability and enhance the physiological activity of native compounds, with combination with other agents, synthetic modification, and adoption of analog and prodrug [39, 40].

Our results observed that RAOSMC stimulation by PDGF-bb induced proliferation and cell cycle progression through intracellular pathways: p42/44 MAPK, p38 MAPK, and Akt cascade, in addition to the activation of PDGFR-*β*. However, PDGF-bb did not induce proliferation and mitogenesis on RAOSMC preincubated with EGCG (Figure 1). Also, pretreated EGCG inhibited the gelatinolytic activity of MMP-9 and conversion from pro-MMP-2 to active MMP-2. Therefore, gelatinolytic activity of MMPs was inhibited dose-dependently in PDGF-bb stimulated RAOSMCs by EGCG (Figure 3). Previous studies reported that EGCG enhanced pro- and active MMP-2 binding to TIMPS and upregulated TIMP-2 expression as one of the major mechanisms for inhibition of SMC invasion [16, 41]. These results suggest that EGCG regulates the activation of MMPs and TIMPs for inhibition of invasion in dedifferentiated VSMCs.

These results suggest that EGCG may mediate the inhibition of PDGF-bb directly binding with PDGFR-*β* on the RAOSMC membrane of RAOSMC and thus deactivate the PDGF signal pathway related to mitogenesis (Figure 4). Some studies reported that EGCG is hijacked by the laminin receptor (LamR), a lipid raft protein, and alters membrane domain composition to prevent epidermal growth factor (EGF) from binding to its receptor (EGFR) [42, 43]. Also, EGCG has been shown to incorporate itself into the plasma membrane to lead to reversible binding of PDGF-bb to a nonreceptor target site, reducing PDGF binding to its receptors [44]. Thus, EGCG inhibits a surface-membrane linked mechanism [45].

In this study, we could also demonstrate on the direct interaction between EGCG and PDGF-bb when they are cotreated. As shown in Figure 2, low concentration of EGCG (10 *μ*M) induces antiproliferation and cell cycle arrest, and cell stimulation occurred in the presence of EGCG. This effect is accompanied by the fact that EGCG inhibits PDGF-induced mitogenesis by disturbing PDGFR-*β* phosphorylation (Figure 5). Also, the inhibitory effect of EGCG was mediated by the blockage of PDGFR-*β* phosphorylation early in the experiment. Thus, EGCG may already have interacted with PDGF-bb in media and inhibited VSMC dedifferentiation by blocking the early signal transduction pathway. Other research groups showed that EGCG is able to interact with various biomolecules, especially proliferation-related proteins, each being proved by various cell line experiments [46–51]. Therefore, recent studies have revealed that EGCG binds with high affinity to residues located in the serum albumin under physiological conditions [52, 53].

Based on our findings, we suggest that EGCG inhibits RAOSMC mitogenesis by interruption of PDGF-bb signaling, probably by blockage of PDGF-bb binding and PDGFR-*β* phosphorylation, as well as the activation of p42/44 MAPK, p38 MAPK, and Akt, important downstream events of PDGFR-*β*. Therefore, EGCG may be a potential target for inhibiting PDGFR and may be of use in the prevention and treatment of vascular diseases.
